# Exploring the roles of urinary HAI-1, EpCAM & EGFR in bladder cancer prognosis & risk stratification

**DOI:** 10.18632/oncotarget.25397

**Published:** 2018-05-18

**Authors:** Kym I.E. Snell, Douglas G. Ward, Naheema S. Gordon, James C. Goldsmith, Andrew J. Sutton, Prashant Patel, Nicholas D. James, Maurice P. Zeegers, K.K. Cheng, Richard T. Bryan

**Affiliations:** ^1^ Centre for Prognosis Research, Research Institute for Primary Care and Health Science, Keele University, Newcastle-under-Lyme, ST5 5BG, UK; ^2^ Institute of Cancer and Genomic Sciences, University of Birmingham, Birmingham B15 2TT, UK; ^3^ Leeds Institute of Health Sciences, University of Leeds and The Diagnostic Evidence Cooperative, Leeds LS2 9JT, UK; ^4^ Institute of Cancer and Genomic Sciences, University of Birmingham and University Hospitals Birmingham NHS Foundation Trust, Birmingham B15 2TH, UK; ^5^ NUTRIM School for Nutrition and Translational Research in Metabolism and CAPHRI Care and Public Health Research Institute, Maastricht University, Maastricht 6200 MD, The Netherlands; ^6^ Institute of Applied Health Research, University of Birmingham, Birmingham B15 2TT, UK

**Keywords:** HAI-1, EpCAM, EGFR, bladder cancer, prognosis

## Abstract

**Objectives:**

To investigate whether elevated urinary HAI-1, EpCAM and EGFR are independent prognostic biomarkers within non-muscle-invasive bladder cancer (NMIBC) patients, and have utility for risk stratification to facilitate treatment decisions.

**Results:**

After accounting for EAU risk group in NMIBC patients, the risk of BC-specific death was 2.14 times higher (95% CI: 1.08 to 4.24) if HAI-1 was elevated and 2.04 times higher (95% CI: 1.02 to 4.07) if EpCAM was elevated. The majority of events occurred in the high-risk NMIBC group and this is where the biggest difference is seen in the survival curves when plotted for EAU risk groups separately. In MIBC patients, being elevated for any of the three biomarkers was significantly associated with BC-specific mortality after accounting for other risk factors, HR = 4.30 (95% CI: 1.85 to 10.03).

**Patients and Methods:**

Urinary levels of HAI-1, EpCAM and EGFR were measured by ELISA in 683 and 175 patients with newly-diagnosed NMIBC and MIBC, respectively, recruited to the Bladder Cancer Prognosis Programme. Associations between biomarkers and progression, BC-specific mortality and all-cause mortality were evaluated using univariable and multivariable Cox regression models, adjusted for European Association of Urology (EAU) NMIBC risk groups. The upper 25% of values for each biomarker within NMIBC patients were considered as elevated. Exploratory analyses in urine from MIBC patients were also undertaken.

**Conclusion:**

Urinary HAI-1 and EpCAM are prognostic biomarkers for NMIBC patients. These biomarkers have potential to guide treatment decisions for high-risk NMIBC patients. Further analyses are required to define the roles of HAI-1, EpCAM and EGFR in MIBC patients.

## INTRODUCTION

High-risk non-muscle-invasive bladder cancer (HR-NMIBC [1]) represents over 30% of all incident bladder cancers [2]. These patients are at considerable risk of progression [1, 3], and 20–30% will die from bladder cancer within 5-years [4–6]. Current guidelines recommend induction and maintenance intravesical BCG or upfront radical cystectomy for highest risk disease [1], treatments with markedly different morbidity, mortality and long-term patient burden. There are no validated biomarkers that can facilitate treatment decisions [7], and the field has been plagued by unstable BCG supply for 6-years [8].

Shedding of the extracellular domains (ectodomains) of transmembrane proteins is recognised as an important cancer-related phenomenon [9–11]. We have previously reported the identification of the shed ectodomains of HAI-1, EpCAM and EGFR in the urine of bladder cancer patients [12–14]; elevated urine levels of the shed ectodomains of EpCAM and EGFR are associated with worse bladder cancer (BC) specific survival [13, 14]. HAI-1 has not previously been investigated as a prognostic biomarker in BC. Mechanistically, ectodomain shedding is mediated by transmembrane proteases which demonstrate substrate specificity and tight regulation [10, 15]. Hence, levels of these ectodomains in urine may represent measurable indicators of disease-specific post-transcriptional phenomena.

The present study explores the associations between urinary levels of HAI-1, EpCAM and EGFR and disease progression and mortality in a cohort of 858 patients (683 NMIBC patients and 175 MIBC patients) from the West Midlands’ Bladder Cancer Prognosis Programme (BCPP [16]) in order to investigate their utility for risk-stratifying NMIBC patients (and HR-NMIBC patients in particular). We included MIBC patients since these tumours share many biological similarities with HR-NMIBC [17], and so any prognostic signal for these biomarkers in HR-NMIBC patients would also be expected in MIBC patients; furthermore, the investigation of the relationship between these biomarkers and mortality in MIBC patients acts as an internal corroboration of findings. These biomarkers were originally discovered within subsets of BCPP patients [12–14], and the current work represents comprehensive repeat ELISA measurements and analysis within all available BCPP urine samples to confirm our preliminary findings using more data, and also to investigate biomarker associations within NMIBC patients specifically.

## RESULTS

### Summary characteristics

**Figure 1:**
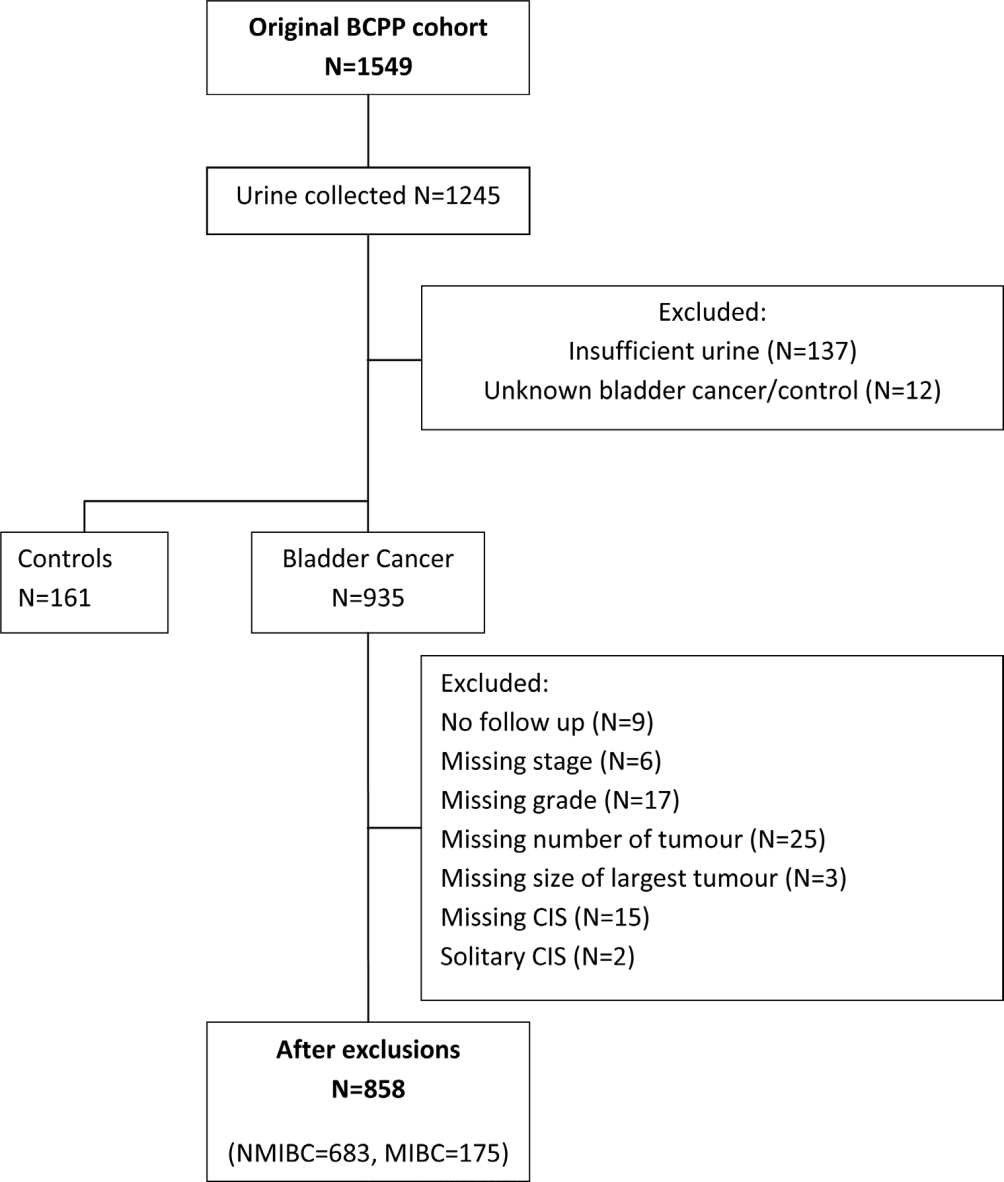
Flow diagram showing exclusions made prior to analysis

After exclusions, there were 858 patients remaining in the dataset, of which 683 were diagnosed with NMIBC and 175 with MIBC (Figure 1). Patient and tumour characteristics for NMIBC and MIBC patients are shown in Table 1. The median age was 71 years and 75 years in NMIBC and MIBC patients, respectively. The proportion of females was 20–21% in both groups. In the NMIBC group, 12% of patients had concomitant CIS, whereas this was higher in the MIBC group (21%). Patients were followed up for a median duration of 4.5 years and 3.8 years in the NMIBC and MIBC groups, respectively. Of all recorded deaths, 27% and 67% were related to BC rather than treatment or other causes in NMIBC and MIBC groups, respectively.

**Table 1:** Patient and tumour characteristics in non-muscle-invasive and muscle-invasive bladder cancer patients

	NMIBC (*N* = 683)	MIBC (*N* = 175)
**Patient demographics**	***n* (%)**	***n* (%)**
Age, years^*^	70.6 [62.9 to 77.3]	75.1 [66.1 to 88.2]
Sex		
Male	540 (79.1)	140 (80.0)
Female	143 (20.9)	35 (20.0)
Smoking		
Smoker	124 (18.2)	35 (20.0)
Ex-smoker	364 (53.3)	109 (62.3)
Never smoked	148 (21.7)	16 (9.1)
Missing	47 (6.9)	15 (8.6)
**Cancer Information**		
Tumour size, cm^*^	2 [1 to 3]	4 [3 to 6]
Grade		
Grade 1	198 (29.0)	0 (0)
Grade 2	260 (38.1)	10 (5.7)
Grade 3	225 (32.9)	165 (94.3)
Stage		
pTa	471 (69.0)	0 (0)
pT1	212 (31.0)	0 (0)
pT2+	0 (0)	175 (100.0)
CIS		
Yes	83 (12.2)	36 (20.6)
No	372 (54.5)	82 (46.9)
Not stated	228 (33.4)	57 (32.6)
**Follow-up**	**Median (95% CI for median)**	**Median (95% CI for median)**
Follow-up duration, years	4.5 (4.3 to 4.7)	3.8 (3.2 to 4.2)
Death^+^		
No	558 (81.7)	47 (26.9)
Yes	125 (18.3)	128 (73.1)
Death related to bladder cancer	34 (27.2)	86 (67.2)
Death related to treatment	1 (0.8)	4 (3.1)
Death related to other causes	89 (71.2)	37 (28.9)
Cause of death missing	1 (0.8)	1 (0.8)
Progression^+^		
Yes	57 (8.3)	-
No	453 (66.3)	-
Missing	173 (25.3)	-

^*^Median [interquartile range] reported for non-normal continuous variable.

^+^Frequency (percentage) for categorical variable.

Of the 858 patients, 496 patients (57.8%), 359 (41.8%) and 387 (45.1%) had been used in previous analyses of urinary EGFR, EpCAM, HAI-1 in BC, respectively [12–14]. The previous analyses had only considered HAI-1 as a diagnostic biomarker, and not as a prognostic biomarker. Here we utilise more patient samples, and all of the assays were run in a single series of replicated experiments to generate a more comprehensive data set.

### Prognostic value of HAI-1, EpCAM & EGFR in NMIBC patients

**Figure 2:**
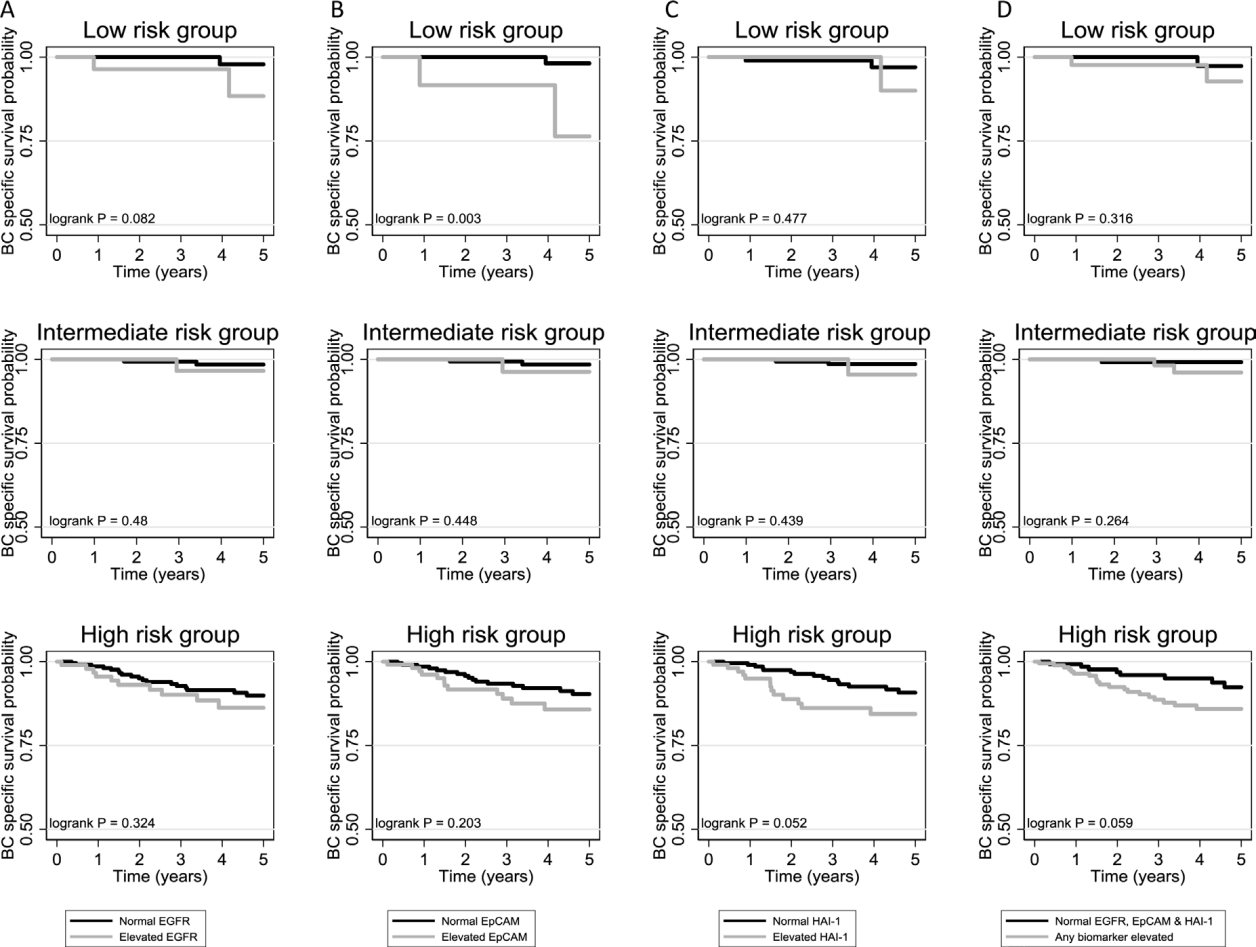
Bladder cancer specific survival curves for normal and elevated biomarker test results for (**A**) EGFR, (**B**) EpCAM, (**C**) HAI-1 and (**D**) combination of all three biomarkers, within each EAU risk group in NMIBC patients.

**Figure 3:**
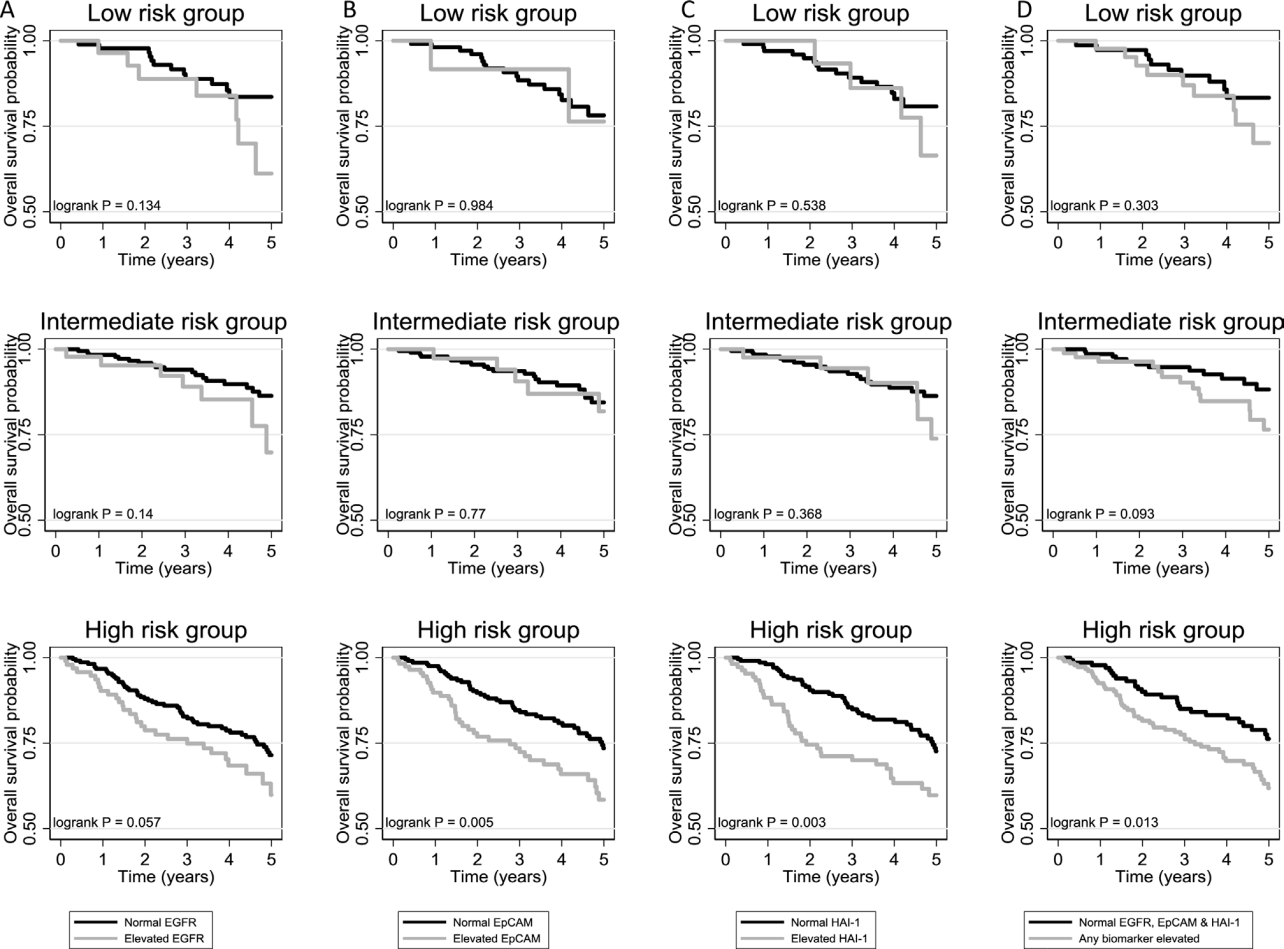
Overall survival curves for normal and elevated biomarker test results for (**A**) EGFR, (**B**) EpCAM, (**C**) HAI-1 and (**D**) combination of all three biomarkers, within each EAU risk group in NMIBC patients.

Using the upper quartile of biomarker values in NMIBC patients, the cut-offs for an elevated biomarker result were defined as 2 ng/mg creatinine, 29 pg/mg creatinine and 392 pg/mg creatinine for HAI-1, EpCAM and EGFR, respectively. The number and proportion of elevated biomarker values across EAU risk groups is shown in Table 2. The unadjusted and adjusted (for EAU risk group) hazard ratios (HRs) from Cox models for each biomarker and for each outcome are shown in Table 3 (full multivariable estimates provided in Supplementary Tables 1–4). Elevated EpCAM and HAI-1 are prognostic for BC-specific death after accounting for EAU risk group, HR = 2.04 (95% CI: 1.02 to 4.07) & HR = 2.14 (95% CI: 1.08 to 4.24), respectively. The majority of events occur in the high-risk NMIBC group and this is where the biggest difference is seen in the survival curves when plotted for EAU risk groups separately (Figure 2). The same is seen for all-cause mortality, although there is more separation between survival curves in high-risk NMIBC (Figure 3). Although the highest adjusted HR observed for BC-specific mortality is 2.41 (95% CI: 1.14 to 5.07) for “any biomarker elevated” (Table 3), the separation between survival curves is similar to that for HAI-1 alone (Figure 2); the unadjusted and adjusted HRs for “any biomarker elevated” are no greater than for HAI-1 when assessing all-cause mortality (Table 3 and Figure 3). There appeared to be no difference in treatment modality utilised between patients with normal and elevated biomarker values (Supplementary Table 5).

**Table 2:** Number and proportion of NMIBC patients in each EAU risk group (and overall) with an elevated biomarker result

	Low risk (*n* = 123)	Intermediate risk (*n* = 237)	High risk (*n* = 323)	Across all risk groups (*n* = 683)
EGFR	28 (22.8%)	46 (19.4%)	96 (29.7%)	170 (24.9%)
EpCAM	13 (10.6%)	43 (18.1%)	114 (35.3%)	170 (24.9%)
HAI-1	18 (14.6%)	43 (18.1%)	110 (34.1%)	171 (25.0%)

**Table 3:** Cox model estimates for biomarkers from univariable (unadjusted) and multivariable models in NMIBC patients (*N* = 683 for BC-specific and all-cause mortality, *N* = 510 for progression)

	BC-specific mortality				All-cause mortality				Progression			
	Unadjusted		Adjusted for EAU risk group		Unadjusted		Adjusted for EAU risk group		Unadjusted		Adjusted for EAU risk group	
	HR^*^ (95% CI)	*P*-value	HR^*^ (95% CI)	*P*-value	HR^*^ (95% CI)	*P*-value	HR^*^ (95% CI)	*P*-value	HR (95% CI)	*P*-value	HR (95% CI)	*P*-value
Elevated EGFR	2.08 (1.03 to 4.19)	0.040	1.76 (0.87 to 3.56)	0.115	1.84 (1.27 to 2.67)	0.001	1.70 (1.17 to 2.48)	0.005	1.16 (0.63 to 2.12)	0.629	1.08 (0.59 to 1.99)	0.798
Elevated EpCAM	2.92 (1.48 to 5.76)	0.002	2.15 (1.08 to 4.29)	0.030	1.94 (1.34 to 2.81)	<0.001	1.71 (1.18 to 2.50)	0.005	1.50 (0.84 to 2.68)	0.166	1.33 (0.74 to 2.40)	0.341
Elevated HAI-1	2.59 (1.31 to 5.12)	0.006	2.15 (1.09 to 4.26)	0.028	1.95 (1.35 to 2.81)	<0.001	1.77 (1.22 to 2.55)	0.002	1.85 (1.08 to 3.20)	0.026	1.69 (0.97 to 2.93)	0.064
Any biomarker elevated	3.05 (1.45 to 6.40)	0.003	2.45 (1.16 to 5.17)	0.019	1.99 (1.39 to 2.86)	<0.001	1.81 (1.26 to 2.60)	0.001	1.80 (1.07 to 3.04)	0.027	1.67 (0.98 to 2.83)	0.058

^*^ Hazard ratios are relative to baseline categories which are normal EGFR, normal EpCAM and normal HAI-1.

**Table 4:** Cox model estimates for biomarkers from univariable (unadjusted) and multivariable models in MIBC patients (*N* = 175)

	BC-specific mortality				All-cause mortality			
	Unadjusted		Adjusted+		Unadjusted		Adjusted+	
	HR^*^ (95% CI)	*P*-value	HR^*^ (95% CI)	*P*-value	HR^*^ (95% CI)	*P*-value	HR^*^ (95% CI)	*P*-value
Elevated EGFR	1.57 (1.01 to 2.44)	0.043	1.62 (1.02 to 2.58)	0.040	1.66 (1.16 to 2.39)	0.006	1.75 (1.19 to 2.56)	0.004
Elevated EpCAM	1.47 (0.95 to 2.25)	0.080	1.38 (0.88 to 2.18)	0.163	1.35 (0.95 to 1.92)	0.089	1.33 (0.92 to 1.93)	0.132
Elevated HAI-1	2.62 (1.62 to 4.23)	<0.001	2.59 (1.58 to 4.23)	<0.001	2.11 (1.45 to 3.08)	<0.001	2.12 (1.44 to 3.13)	<0.001
Any biomarker elevated	4.40 (1.90 to 10.17)	0.001	4.30 (1.85 to 10.03)	0.001	3.06 (1.68 to 5.58)	<0.001	3.07 (1.67 to 5.63)	<0.001

^*^Hazard ratios are relative to baseline categories which are EAU low risk, normal EGFR, normal EpCAM and normal HAI-1.

^+^Adjusted for grade, size of tumour, single or multiple tumours and CIS.

The association of elevated biomarker levels with the risk of progression from NMIBC to MIBC is weaker than for death, HAI-1 HR = 1.85 (95% CI: 1.07 to 3.20) unadjusted and HR = 1.69 (95% CI: 0.97 to 2.93) adjusted for EAU risk group; neither EpCAM nor EGFR reached significance (Table 3). The progression-free survival curves according to each biomarker in each risk group are shown in Supplementary Figure 1.

### Prognostic value of HAI-1, EpCAM & EGFR in MIBC patients

A higher proportion of patients had elevated biomarkers in the MIBC group with 61.7% elevated for HAI-1, 53.1% elevated for EpCAM and 58.3% elevated for EGFR. To maintain uniformity with the NMIBC analyses, we used individual components of the EAU risk stratification for multivariable analyses: grade, tumour size and multiplicity, and the presence or absence of CIS (all patients newly-diagnosed, stages T2+) [3]. Since these factors are not entirely relevant for prognostication in MIBC, these represent exploratory analyses only. Multivariable Cox models adjusting for these risk factors suggest HAI-1 and EGFR to be independent prognostic factors for BC-specific mortality (Table 4; full multivariable models in Supplementary Tables 6–9). Being elevated for any of the three biomarkers was significantly associated with BC-specific mortality after accounting for other risk factors, HR = 4.30 (95% CI: 1.85 to 10.03), with the survival curves showing early separation (Supplementary Figure 2).

## DISCUSSION

We have demonstrated that elevated levels of urinary HAI-1 and EpCAM are associated with BC-specific mortality in high-risk NMIBC patients: each biomarker is associated with increased risk of death from disease within 5-years (adjusted for EAU risk group). Exploratory analyses demonstrate an association between elevated urinary HAI-1 and EGFR and increased risk of death in MIBC patients. For HAI-1, these associations have not been reported previously.

The functions and biology of EpCAM and EGFR have been described elsewhere [12–14]. HAI-1 is a membrane-associated Kunitz-type serine proteinase inhibitor that inhibits hepatocyte growth factor activator, matriptase, and other members of the S1 family of trypsin-like serine proteases. The mechanisms of ectodomain shedding, and the interplay of membrane-bound proteases and their regulatory molecules [15], add an extra layer of complexity to the molecular pathology of BC [14]. Thus, measuring HAI-1, EpCAM and EGFR in urine samples collected at the time of diagnosis (after diagnostic cystoscopy, prior to TURBT) represents assessment of biological processes associated with BC pathogenesis, and the subsequent risk of disease-specific death. Given that we demonstrated a weaker association for NMIBC progression (to MIBC) than for mortality, it is likely that these processes predominate later in disease pathogenesis, and are possibly related to the development of metastases. This is supported by the observation that these proteins remain prognostic when measured in the urine of patients already diagnosed with MIBC.

Despite extensive efforts at the genomic, epigenomic and transcriptomic analysis of high-risk NMIBC, no validated tissue-based prognostic biomarkers have emerged. We suggest that urinary HAI-1 and EpCAM could be used to facilitate decision-making regarding the treatment of patients with high-risk NMIBC – a simple stratification tool, with an elevated test guiding patients and clinicians towards more radical therapy instead of bladder preservation. Furthermore, recruiting and randomising BC patients to trials of bladder preservation versus radical cystectomy is notoriously difficult [18]. In these settings, one should have the strongest rationale possible (i.e. death from disease) to counsel or stratify patients towards one treatment modality or the other. Urinary biomarkers such as HAI-1 and EpCAM may be useful tools for facilitating such patient counselling, recruitment and randomisation.

For MIBC, the clinical utility of our biomarkers for patient and treatment stratification is less obvious. We caution against over-interpretation since these were exploratory analyses; we would have liked to adjust for more appropriate risk factors had they been available, but BCPP is a NMIBC-focused study [16]. However, these data do align with our findings in high-risk NMIBC, as discussed above.

We appreciate that there are limitations to this study. We chose an arbitrary cutpoint to define an elevated test result (upper quartile); however, we felt that this approximated to the proportion of UK patients who undergo cystectomy as the initial primary treatment of high-risk NMIBC (c.25%). The number of progression events and BC-deaths were also low, resulting in low power to detect associations. However, although not always statistically significant, the direction of associations was consistent for the biomarkers with each of the outcomes, and within NMIBC and MIBC groups. Also due to the low number of events, we could not include many variables in the models individually and decided to simply adjust for EAU NMIBC risk group. Future work in larger cohorts with more events should additionally adjust for other potential confounders. In addition, treatment data for high-risk NMIBC patients were reported inconsistently and we could not analyse them reliably, nor incorporate the data into multivariable analyses; however, a simple observational analysis using the data that were recorded suggested similar proportions of normal and elevated biomarker levels within each treatment category and a low probability of influencing the biomarker relationships observed. Clearly, validation in an independent cohort is required as the next step of translation, alongside further work to determine a suitable biomarker cut-off value that assists treatment decisions.

Furthermore, since patients’ biospecimens were only collected at the time of diagnosis, it remains unknown whether these biomarkers also have prognostic utility when applied at the time of NMIBC recurrence or progression. Finally, further evaluation of these biomarkers is required in MIBC patients as the clinical utility is less obvious than for NMIBC.

## PATIENTS AND METHODS

### Patients and biospecimens

Urine samples were obtained from the prospective BCPP biospecimen collection (ethics approval 06/MRE04/65) [16]. Patients were recruited between 2005 and 2011 from nine hospitals in the West Midlands region, and gave informed consent for enrolment into BCPP on the basis of initial cystoscopic findings suggestive of primary BC. All patients were newly-diagnosed, had not received BC treatment prior to biospecimen collection, and were subsequently treated and followed-up according to contemporary guidelines (including re-resection where indicated). Inclusion and exclusion criteria are detailed elsewhere [16].

Urine samples were obtained prior to transurethral resection of bladder tumour(s) (TURBT) specifically for biomarker analyses [16]. Samples were placed on ice, centrifuged at 2000 rpm for 10 minutes within 8 hours of collection, and supernatants and pellets separated and stored at −80° C.

Tumour grade and stage records were amended according to results of re-resection or cystectomy (where performed). We used the 1973 grade classification as it was in universal use in the UK at the time of patient recruitment, and is also the basis for the EORTC and EAU NMIBC risk tables [1, 3]. Diagnostic formalin-fixed paraffin-embedded tumour samples were retrieved from local histopathology departments, and 10% of all such samples underwent expert pathological review as part of routine quality assurance. All included tumours were purely or predominantly transitional cell carcinomas.

### ELISAs

ELISAs were performed as previously described [12–14]. Briefly, 20–50 μl of urine supernatant was diluted in phosphate buffered saline containing 1% bovine serum albumin and analysed using DuoSet sandwich ELISAs (R&D Systems, catalogue numbers DY231, DY960, DY1048). Urine samples were randomly assigned to ELISA plates and QA samples used to verify the consistency of results across plates. Urinary protein concentrations were normalised to urinary creatinine.

### Statistical analyses

Our previous work analysed urine samples for HAI-1, EpCAM and EGFR levels in subsets of patients [12–14]. In the current study, we repeated the analyses of all 3 biomarkers in more patients using data from a new series of biomarker assays conducted in duplicate. Figure 1 shows the exclusions made and final number of individuals used in the analysis.

Individuals were excluded from analysis if they were determined to not have bladder cancer or it was unknown. A complete case analysis was used; therefore, individuals were also excluded if they did not have biomarker measurements, were missing follow-up information, or were missing risk factor information for stage, grade, size, number of tumours, or presence or absence of CIS. Individuals with solitary CIS were excluded. Biomarkers were measured in all patients who provided a sufficient volume of urine for analysis; however, patient characteristics were compared between patients with and without biomarker measurements (Supplementary Table 10).

Three outcomes were considered: progression to MIBC (for NMIBC patients), bladder cancer specific mortality and all-cause mortality. Survival times were calculated from the date of registration into the BCPP study until the date of progression, date of death (for all-cause mortality), or date of death from bladder cancer (for bladder cancer specific mortality). Individuals were censored at the date last known to be progression-free (for progression), alive, or additionally at the date of death from other causes for bladder cancer specific mortality.

Patient characteristics were summarised using frequencies and percentages for categorical variables and medians with interquartile ranges (IQR) for skewed continuous variables. Follow-up was summarised using the median duration and 95% confidence interval (CI) for the median.

For each biomarker, the cut-off for an elevated result was set at the 75th percentile of all values within NMIBC patients, therefore fixing 25% of NMIBC patients to be ‘elevated’ and approximating to the proportion of UK patients who undergo cystectomy as the initial primary treatment of high-risk NMIBC. Patients were categorised into EAU risk groups (low, intermediate and high) [1]. Kaplan-Meier survival curves were plotted to compare normal and elevated values for each biomarker in all NMIBC patients, as well as within each EAU risk group. Univariable Cox proportional hazards models were fitted to estimate the associations between each biomarker (elevated versus normal) and outcome (either progression, bladder cancer specific mortality or all-cause mortality). Biomarkers were also investigated in combination (if any of EGFR, EpCAM or HAI-1 were elevated). Additionally, multivariable Cox models were fitted, adjusting for EAU risk group. As patients were recruited from multiple centres, all Cox models included a shared frailty term to account for within-centre clustering. The proportional hazards assumption was checked using log-log plots for all variables included in any of the models and for all outcomes. Hazard ratios and 95% CIs are reported for each biomarker, including both unadjusted and adjusted (for EAU risk group) results.

The biomarkers were also evaluated in MIBC patients using Kaplan-Meier plots and Cox models as for NMIBC patients. However, EAU risk groups are specific to NMIBC patients; therefore, in the multivariable models, adjustment was made for the individual factors that make up the EAU risk groups: stage, grade, size, number of tumours and presence or absence of CIS.

Principal treatment was summarised for the high-risk NMIBC group using frequencies and percentages for normal and elevated values for each biomarker.

Statistical analyses were performed using Stata MP 14.0 (StataCorp, College Station, TX, USA).

## CONCLUSIONS

Elevated levels of urinary HAI-1 and EpCAM are independent prognostic factors for BC-specific survival in NMIBC patients, and may be useful for informing both clinician and patient decisions regarding the initial management of HR-NMIBC by primary cystectomy or bladder-preservation.

## SUPPLEMENTARY MATERIALS FIGURES AND TABLES



## Abbreviations

BC: bladder cancer
BCPP: Bladder Cancer Prognosis Programme (West Midlands, UK)
CI: confidence interval
CIS: carcinoma *in situ*
EAU: European Association of Urology
EGFR: epidermal growth factor receptor
ELISA: enzyme-linked immunosorbent assay
EORTC: European Organisation for Research and Treatment of Cancer
EpCAM: epithelial cell adhesion molecule
HAI-1: hepatocyte growth factor activator inhibitor-1
HR: hazard ratio
HR-NMIBC: high-risk non-muscle-invasive bladder cancer
MIBC: muscle-invasive bladder cancer
NMIBC: non-muscle-invasive bladder cancer
QA: quality assurance
TURBT: transurethral resection of bladder tumour(s)

## References

[1] Babjuk M, Bohle A, Burger M, Capoun O, Cohen D, Comperat EM, Hernandez V, Kaasinen E, Palou J, Roupret M, van Rhijn BW, Shariat SF, Soukup V (2017). EAU Guidelines on Non-Muscle-invasive Urothelial Carcinoma of the Bladder: Update 2016. Eur Urol.

[2] Boustead GB, Fowler S, Swamy R, Kocklebergh R, Hounsome L (2014). Stage, grade and pathological characteristics of bladder cancer in the UK: British Association of Urological Surgeons (BAUS) urological tumour registry. BJU Int.

[3] Sylvester RJ, van der Meijden AP, Oosterlinck W, Witjes JA, Bouffioux C, Denis L, Newling DW, Kurth K (2006). Predicting recurrence and progression in individual patients with stage Ta T1 bladder cancer using EORTC risk tables: a combined analysis of 2596 patients from seven EORTC trials. Eur Urol.

[4] Noon AP, Albertsen PC, Thomas F, Rosario DJ, Catto JW (2013). Competing mortality in patients diagnosed with bladder cancer: evidence of undertreatment in the elderly and female patients. Br J Cancer.

[5] Thomas F, Rosario DJ, Rubin N, Goepel JR, Abbod MF, Catto JW (2012). The long-term outcome of treated high-risk nonmuscle-invasive bladder cancer: time to change treatment paradigm?. Cancer.

[6] Thomas F, Noon AP, Rubin N, Goepel JR, Catto JW (2013). Comparative outcomes of primary, recurrent, and progressive high-risk non-muscle-invasive bladder cancer. Eur Urol.

[7] Kamat AM, Vlahou A, Taylor JA, Hudson ML, Pesch B, Ingersoll MA, Todenhofer T, van RB, Kassouf W, Barton GH, Behrens T, Chandra A, Goebell PJ (2014). Considerations on the use of urine markers in the management of patients with high-grade non-muscle-invasive bladder cancer. Urol Oncol.

[8] Mostafid AH, Palou RJ, Sylvester R, Witjes JA (2015). Therapeutic options in high-risk non-muscle-invasive bladder cancer during the current worldwide shortage of bacille Calmette-Guerin. Eur Urol.

[9] Hansen AG, Freeman TJ, Arnold SA, Starchenko A, Jones-Paris CR, Gilger MA, Washington MK, Fan KH, Shyr Y, Beauchamp RD, Zijlstra A (2013). Elevated ALCAM shedding in colorectal cancer correlates with poor patient outcome. Cancer Res.

[10] Hartmann M, Parra LM, Ruschel A, Bohme S, Li Y, Morrison H, Herrlich A, Herrlich P (2015). Tumor Suppressor NF2 Blocks Cellular Migration by Inhibiting Ectodomain Cleavage of CD44. Mol Cancer Res.

[11] Higashiyama S, Nanba D, Nakayama H, Inoue H, Fukuda S (2011). Ectodomain shedding and remnant peptide signalling of EGFRs and their ligands. J Biochem.

[12] Shimwell NJ, Bryan RT, Wei W, James ND, Cheng KK, Zeegers MP, Johnson PJ, Martin A, Ward DG (2013). Combined proteome and transcriptome analyses for the discovery of urinary biomarkers for urothelial carcinoma. Br J Cancer.

[13] Bryan RT, Shimwell NJ, Wei W, Devall AJ, Pirrie SJ, James ND, Zeegers MP, Cheng KK, Martin A, Ward DG (2014). Urinary EpCAM in urothelial bladder cancer patients: characterisation and evaluation of biomarker potential. Br J Cancer.

[14] Bryan RT, Regan HL, Pirrie SJ, Devall AJ, Cheng KK, Zeegers MP, James ND, Knowles MA, Ward DG (2015). Protein shedding in urothelial bladder cancer: prognostic implications of soluble urinary EGFR and EpCAM. Br J Cancer.

[15] Matthews AL, Noy PJ, Reyat JS, Tomlinson MG (2017). Regulation of A disintegrin and metalloproteinase (ADAM) family sheddases ADAM10 and ADAM17: The emerging role of tetraspanins and rhomboids. Platelets.

[16] Zeegers MP, Bryan RT, Langford C, Billingham L, Murray P, Deshmukh NS, Hussain S, James N, Wallace DM, Cheng KK (2010). The West Midlands Bladder Cancer Prognosis Programme: rationale and design. BJU Int.

[17] Czerniak B, Dinney C, McConkey D (2016). Origins of Bladder Cancer. Annu Rev Pathol.

[18] Huddart RA, Hall E, Lewis R, Birtle A (2010). Life and death of spare (selective bladder preservation against radical excision): reflections on why the spare trial closed. BJU Int.

