# Supplementary material for: Exploring the roles of urinary HAI-1, EpCAM & EGFR in bladder cancer prognosis & risk stratification
Source: Oncotarget. 2018 May 18;9(38):25244–53. doi: 10.18632/oncotarget.25397 (PMC5982738; doi:10.18632/oncotarget.25397)
Supplement: Supplementary file 1 [file oncotarget-09-25244-s001.pdf]

Exploring the roles of urinary HAI-1, EpCAM & EGFR in bladder cancer prognosis & risk stratification

SUPPLEMENTARY MATERIALS

Supplementary Table 1: Multivariable Cox model estimates including EGFR in NMIBC patients (*N* = 683 for BC-specific and all-cause mortality, *N* = 510 for progression)

Variables	BC Specific mortality		All-cause mortality		Progression	
	HR* (95% CI)	*P*-value	HR* (95% CI)	*P*-value	HR* (95% CI)	*P*-value
Elevated EGFR	1.76 (0.87 to 3.56)	0.115	1.70 (1.17 to 2.48)	0.005	1.08 (0.59 to 1.99)	0.798
EAU intermediate risk	0.50 (0.10 to 2.5252)	0.404	0.68 (0.37 to 1.24)	0.206	1.07 (0.46 to 2.49)	0.884
EAU high risk	3.64 (1.10 to 12.01)	0.034	1.59 (0.96 to 2.63)	0.070	1.93 (0.89 to 4.18)	0.096

*Hazard ratios are relative to baseline categories which are EAU low risk and normal EGFR.

Supplementary Table 2: Multivariable Cox model estimates including EpCAM in NMIBC patients (*N* = 683 for BC-specific and all-cause mortality, *N* = 510 for progression)

Variables	BC Specific mortality		All-cause mortality		Progression	
	HR* (95% CI)	*P*-value	HR* (95% CI)	*P*-value	HR* (95% CI)	*P*-value
Elevated EpCAM	2.15 (1.08 to 4.29)	0.030	1.71 (1.18 to 2.50)	0.005	1.33 (0.74 to 2.40)	0.341
EAU intermediate risk	0.48 (0.10 to 2.41)	0.375	0.65 (0.36 to 1.18)	0.158	1.05 (0.45 to 2.45)	0.918
EAU high risk	3.21 (0.96 to 10.75)	0.058	1.47 (0.88 to 2.45)	0.136	1.84 (0.84 to 4.01)	0.127

*Hazard ratios are relative to baseline categories which are EAU low risk and normal EpCAM.

Supplementary Table 3: Multivariable Cox model estimates including HAI-1 in NMIBC patients (*N* = 683 for BC-specific and all-cause mortality, *N* = 510 for progression)

Variables	BC Specific mortality		All-cause mortality		Progression	
	HR* (95% CI)	*P*-value	HR* (95% CI)	*P*-value	HR* (95% CI)	*P*-value
Elevated HAI-1	2.15 (1.09 to 4.26)	0.028	1.77 (1.22 to 2.55)	0.002	1.69 (0.97 to 2.93)	0.064
EAU intermediate risk	0.51 (0.10 to 2.52)	0.406	0.67 (0.37 to 1.22)	0.187	1.07 (0.46 to 2.50)	0.878
EAU high risk	3.55 (1.07 to 11.75)	0.038	1.54 (0.93 to 2.55)	0.094	1.81 (0.83 to 3.93)	0.135

*Hazard ratios are relative to baseline categories which are EAU low risk and normal HAI-1.

Supplementary Table 4: Multivariable Cox model estimates including 'any biomarker elevated' in NMIBC patients (*N* = 683 for BC-specific and all-cause mortality, *N* = 510 for progression)

Variables	BC Specific mortality		All-cause mortality		Progression	
	HR* (95% CI)	*P*-value	HR* (95% CI)	*P*-value	HR* (95% CI)	*P*-value
Any biomarker elevated	2.45 (1.16 to 5.17)	0.019	1.81 (1.26 to 2.60)	0.001	1.67 (0.98 to 2.83)	0.058
EAU intermediate risk	0.50 (0.10 to 2.50)	0.400	0.67 (0.37 to 1.22)	0.187	1.09 (0.46 to 2.54)	0.848
EAU high risk	3.34 (1.01 to 11.06)	0.049	1.51 (0.91 to 2.49)	0.112	1.83 (0.84 to 3.97)	0.127

*Hazard ratios are relative to baseline categories which are EAU low risk and all biomarkers normal.

Supplementary Table 5: Principal treatments received by the 323 HR-NMIBC patients

N (%) of treatment group	EGFR		EpCAM		HAI-1		Combination	
	Normal	Elevated	Normal	Elevated	Normal	Elevated	All Normal	Any Elevated
Cystectomy	12 (5)	8 (8)	13 (6)	7 (6)	13 (6)	7 (6)	9 (6)	11 (6)
Course of intravesical BCG	120 (53)	44 (46)	102 (49)	62 (54)	108 (51)	56 (51)	67 (48)	97 (53)
Course of intravesical chemotherapy	18 (8)	6 (6)	18 (9)	6 (5)	15 (7)	9 (8)	11 (8)	13 (7)
Other	33 (15)	13 (14)	35 (17)	11 (10)	33 (15)	13 (12)	25 (18)	21 (11)
Missing	44 (19)	25 (26)	41 (20)	28 (25)	44 (21)	25 (23)	27 (19)	42 (23)
TOTAL	227 (100)	96 (100)	209 (100)	114 (100)	213 (100)	110 (100)	139 (100)	184 (100)

Supplementary Table 6: Multivariable Cox model estimates including EGFR for bladder cancer specific and all-cause mortality in MIBC patients (*N* = 175)

Variables	BC specific mortality		All-cause mortality	
	HR (95% CI)	*P*-value	HR (95% CI)	*P*-value
Elevated EGFR	1.62 (1.02 to 2.58)	0.040	1.75 (1.19 to 2.56)	0.004
Grade 3	1.15 (0.41 to 3.22)	0.794	1.16 (0.50 to 2.69)	0.733
Tumour size ≥3 cm	1.55 (0.84 to 2.85)	0.157	1.36 (0.84 to 2.18)	0.209
Multiple tumours	0.90 (0.56 to 1.45)	0.671	0.88 (0.60 to 1.30)	0.531
CIS	1.01 (0.55 to 1.85)	0.983	1.15 (0.71 to 1.85)	0.580

*Hazard ratios are relative to baseline categories which are normal EGFR, Grade 2, tumour size <3 cm, single tumour and unknown/no CIS.

Supplementary Table 7: Multivariable Cox model estimates including EpCAM for bladder cancer specific and all-cause mortality in MIBC patients (*N* = 175)

Variables	BC specific mortality		All-cause mortality	
	HR (95% CI)	*P*-value	HR (95% CI)	*P*-value
Elevated EpCAM	1.38 (0.88 to 2.18)	0.163	1.33 (0.92 to 1.93)	0.132
Grade 3	1.09 (0.39 to 3.07)	0.864	1.11 (0.48 to 2.57)	0.814
Tumour size ≥3 cm	1.26 (0.67 to 2.37)	0.473	1.10 (0.67 to 1.81)	0.700
Multiple tumours	0.84 (0.52 to 1.35)	0.469	0.82 (0.56 to 1.21)	0.318
CIS	0.88 (0.49 to 1.57)	0.657	0.97 (0.61 to 1.53)	0.885

*Hazard ratios are relative to baseline categories which are normal EpCAM, Grade 2, tumour size <3 cm, single tumour and unknown/no CIS.

Supplementary Table 8: Multivariable Cox model estimates including HAI-1 for bladder cancer specific and all-cause mortality in MIBC patients (*N* = 175)

Variables	BC specific mortality		All-cause mortality	
	HR (95% CI)	*P*-value	HR (95% CI)	*P*-value
Elevated HAI-1	2.59 (1.58 to 4.23)	<0.001	2.12 (1.44 to 3.13)	<0.001
Grade 3	1.44 (0.51 to 4.10)	0.495	1.39 (0.59 to 3.26)	0.454
Tumour size ≥3 cm	1.11 (0.60 to 2.06)	0.747	1.00 (0.63 to 1.63)	0.988
Multiple tumours	0.87 (0.54 to 1.39)	0.559	0.85 (0.58 to 1.25)	0.417
CIS	0.89 (0.50 to 1.59)	0.694	0.97 (0.61 to 1.54)	0.909

*Hazard ratios are relative to baseline categories which are normal HAI-1, Grade 2, tumour size <3cm, single tumour and unknown/no CIS.

Supplementary Table 9: Multivariable Cox model estimates including ‘any biomarker elevated’ for bladder cancer specific and all-cause mortality in MIBC patients (*N* = 175)

Variables	BC specific mortality		All-cause mortality	
	HR (95% CI)	*P*-value	HR (95% CI)	*P*-value
Any biomarker elevated	4.30 (1.85 to 10.03)	0.001	3.07 (1.67 to 5.63)	<0.001
Grade 3	1.15 (0.41 to 3.22)	0.793	1.15 (0.49 to 2.67)	0.748
Tumour size ≥3 cm	1.29 (0.71 to 2.36)	0.408	1.13 (0.71 to 1.81)	0.605
Multiple tumours	0.84 (0.52 to 1.35)	0.471	0.83 (0.56 to 1.22)	0.335
CIS	1.00 (0.56 to 1.80)	1.000	1.08 (0.68 to 1.71)	0.756

*Hazard ratios are relative to baseline categories which are all biomarkers normal, Grade 2, tumour size <3 cm, single tumour and unknown/no CIS.

Supplementary Table 10: Comparison of patient characteristics of those with and without biomarker measurements (after other exclusions have been applied)

	Biomarkers measured (*N* = 858)	Biomarkers missing (*N* = 300)	*P*-value+
**Patient demographics**	***n* (%)**	***n* (%)**	
Age, years*	70.1 [63.5 to 77.9]	71.0 [64.6 to 78.3]	0.243
Gender			0.077
Male	680 (79.3)	223 (74.3)	
Female	178 (20.7)	77 (25.7)	
Smoking			0.001
Smoker	159 (18.5)	65 (21.7)	
Ex-smoker	473 (55.1)	135 (45.0)	
Never smoked	164 (19.1)	59 (19.7)	
Missing	62 (7.2)	41 (13.7)	
**Cancer Information**			
Tumour size, cm*	2 [1.5 to 4]	3 [1.9 to 4]	0.002
Grade			0.118
Grade 1	198 (23.1)	59 (19.7)	
Grade 2	270 (31.5)	84 (28.0)	
Grade 3	390 (45.5)	157 (52.3)	
Stage			0.120
pTa	471 (54.9)	144 (48.0)	
pT1	212 (24.7)	85 (28.3)	
pT2+	175 (20.4)	71 (23.7)	
CIS			0.283
Yes	119 (13.9)	48 (16.0)	
No	454 (52.9)	143 (47.7)	
Not stated	285 (33.2)	109 (36.3)	

*Median [interquartile range] reported for non-normal continuous variable.
+Wilcoxon rank-sum test used for continuous variables and chi-squared test used for categorical variables.

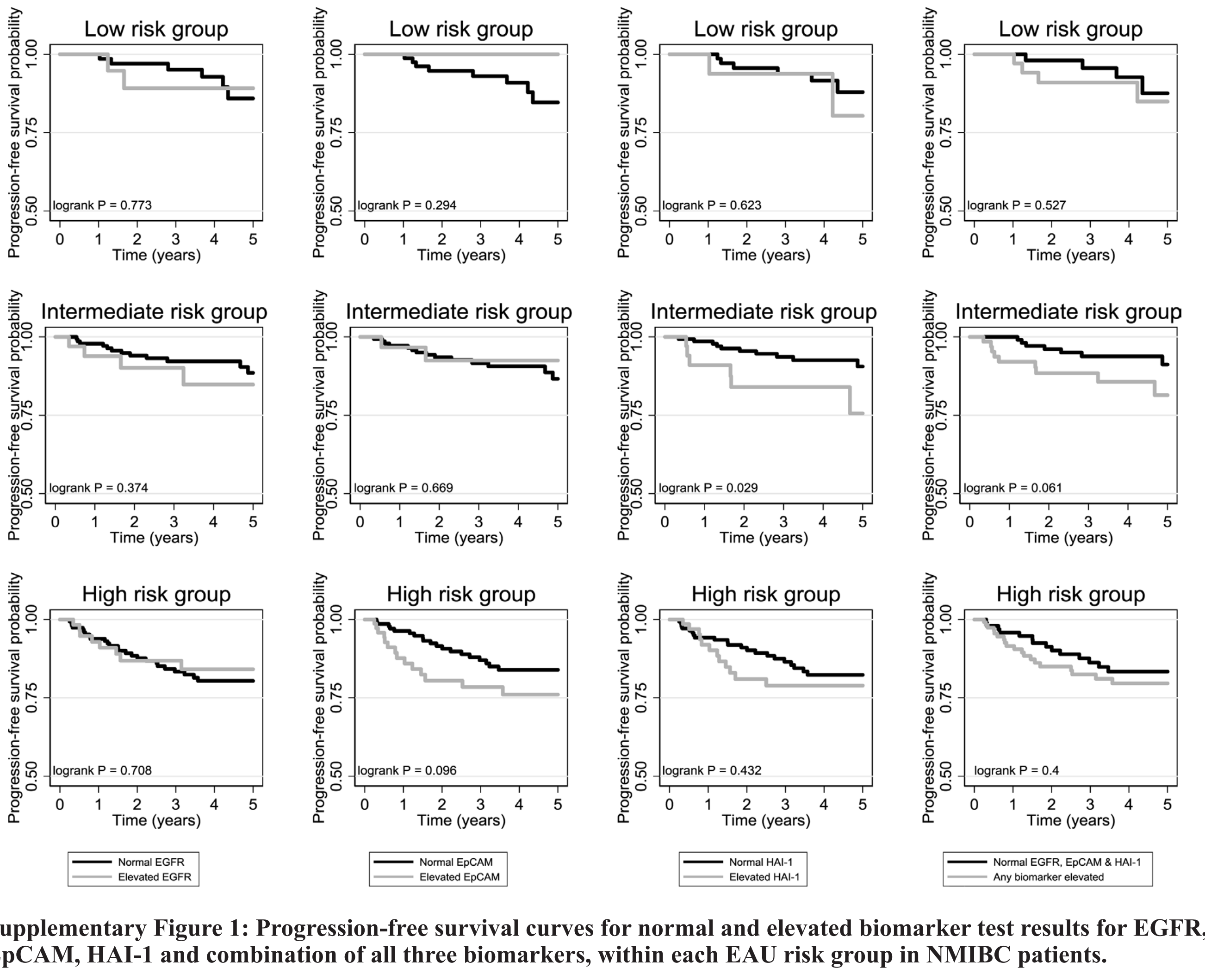

Supplementary Figure 1: Progression-free survival curves for normal and elevated biomarker test results for EGFR, EpCAM, HAI-1 and combination of all three biomarkers, within each EAU risk group in NMIBC patients.

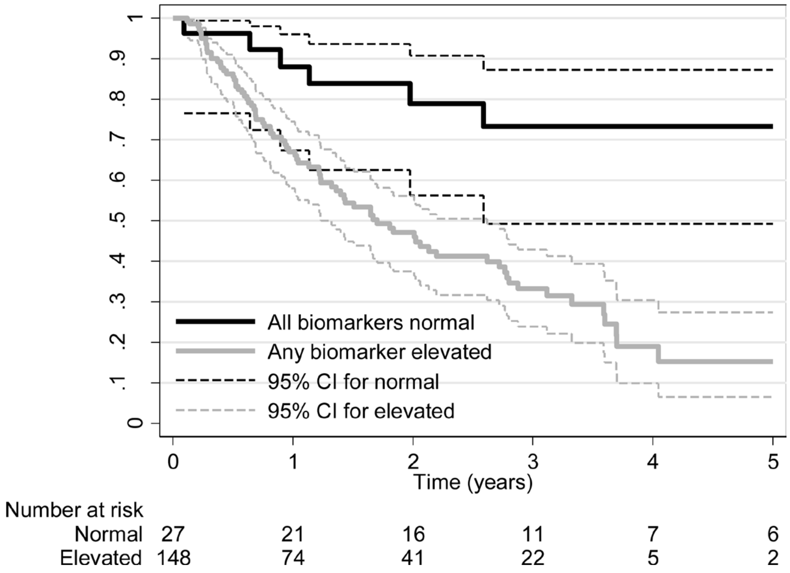

Supplementary Figure 2: Bladder cancer specific survival curves for combination of all 3 biomarker test results in MIBC patients.
